# PPAR Medicines and Human Disease: The ABCs of It All

**DOI:** 10.1155/2012/504918

**Published:** 2012-08-07

**Authors:** Anthony J. Apostoli, Christopher J. B. Nicol

**Affiliations:** ^1^Department of Pathology and Molecular Medicine, Queen's University, Kingston, ON, Canada K7L 3N6; ^2^Cancer Biology and Genetics Division, Cancer Research Institute, Queen's University, Kingston, ON, Canada K7L 3N6; ^3^Department of Biomedical and Molecular Sciences, Queen's University, Kingston, ON, Canada K7L 3N6

## Abstract

ATP-dependent binding cassette (ABC) transporters are a family of transmembrane proteins that pump a variety of hydrophobic compounds across cellular and subcellular barriers and are implicated in human diseases such as cancer and atherosclerosis. Inhibition of ABC transporter activity showed promise in early preclinical studies; however, the outcomes in clinical trials with these agents have not been as encouraging. Peroxisome proliferator-activated receptors (PPARs) are ligand-activated transcription factors that regulate genes involved in fat and glucose metabolism, and inflammation. Activation of PPAR signaling is also reported to regulate ABC gene expression. This suggests the potential of PPAR medicines as a novel means of controlling ABC transporter activity at the transcriptional level. This paper summarizes the advances made in understanding how PPAR medicines affect ABC transporters, and the potential implications for impacting on human diseases, in particular with respect to cancer and atherosclerosis.

## 1. Introduction

Harnessing the energy released from adenosine triphosphate (ATP) hydrolysis, ATP-dependent binding cassette (ABC) transporters shuttle a wide range of substrates, including lipids, metabolites, and xenobiotics, across biological membranes in order to maintain normal cell metabolism. They represent the largest family of transmembrane proteins in humans, comprising 49 ABC genes, and are best reviewed elsewhere [1–3]. These genes are subdivided among seven subfamilies (A-G) based on sequence and structural homology and are highly conserved among eukaryotic species, suggesting that most appeared early in metazoan evolution [4]. The proteins encoded by ABC genes consist of two distinct domains: a transmembrane domain that recognizes specific compounds and transports them across cellular and subcellular barriers and a nucleotide-binding domain where ATP hydrolysis occurs to yield energy for substrate transport [5]. Typically, ABC proteins are unidirectional transporters expressed at the cell membrane, which move hydrophobic molecules internally for metabolic pathways, or externally for elimination from the cell and/or use by other tissues and organs. Thus, ABC transporters play important roles in a range of human physiologic, toxicologic, and pathologic functions. With respect to the latter, many preclinical reports that show promise in terms of regulating ABC transporters to overcome chemotherapeutic drug resistance in tumours, or modify lipid homeostasis in order to reduce atherosclerotic risk, have not achieved the same level of success in clinical trials.

Peroxisome proliferator-activated receptors (PPARs) are ligand-activated transcription factors that regulate expression of a plethora of genes involved in sugar and fat metabolism, inflammation, and cancer [6–8]. Three PPAR homologs have been characterized—PPAR*α*, PPAR*β*/*δ*, and PPAR*γ*—each displaying a unique pattern of tissue-specific expression that reflect their distinctive functions [9–11]. Recently, there is mounting *in vitro* and *in vivo* evidence that activation of PPARs may alter ABC protein expression and/or function. Accordingly, this paper will summarize recent developments in an emerging field where PPAR medicines, capable of modulating ABC transporter genes at the transcriptional level, may prove useful when such modulation provides novel therapeutic options for treating cancer and atherosclerosis.

## 2. PPARs and Their Ligands

As members of the nuclear receptor superfamily, PPARs contain a ligand-binding domain that recognizes and binds specific PPAR agonists, and a DNA-binding domain that interacts with specific peroxisome proliferator-response elements (PPREs) within the genome [12]. PPARs are localized to the nucleus and dimerize with retinoid *X* receptor (RXR)*α* to form complexes that bind to PPREs in the promoter regions of a broad range of target genes [13]. In its resting state, the PPAR : RXR*α* complex associates with cell-specific corepressor molecules that aid in the silencing of target gene transcription. Ligand binding elicits a conformational change in PPAR that leads to the release of corepressors, and the recruitment of coactivator molecules that promote target gene transcriptional activity. Furthermore, ligand activation of PPARs may also repress signaling of some gene targets through direct interaction with other transcription factors or competition for available coregulators [14].

PPAR*α* is highly expressed in the liver, heart, kidney, skeletal muscle, and large intestine [15]. It is activated by the “fibrate” class of drugs, such as bezafibrate, ciprofibrate, clofibrate, gemfibrozil, and fenofibrate, used to treat elevated triglycerides and low high-density lipoprotein (HDL) [16]. PPAR*β*/*δ* is more ubiquitously expressed with highest levels noted within the large intestine and placenta [15]. Similar to other PPAR subtypes, it may also be activated by various saturated and unsaturated fatty acids [12]. Because less is understood about PPAR*β*/*δ*, fewer synthetic activators have been developed; however, emerging evidence supports the potential therapeutic value of PPAR*β*/*δ* agonists, such as GW0742, GW501516, and MBX-8025, which remain to be clinically tested [17].

As a chief regulator of adipogenesis, PPAR*γ* is abundantly expressed in adipose tissue [18], and like PPAR*α*, is also detected in vascular and immune cells, as well as tissues such as the colon, breast, and prostate [19, 20]. Synthetic agents known as thiazolidinediones (TZDs) like troglitazone, ciglitazone, rosiglitazone, and pioglitazone are classic examples of PPAR*γ* activators [21]. In North America, rosiglitazone and pioglitazone are still prescribed to treat type 2 diabetic patients. However, there are reports suggesting increased myocardial infarction risk with rosiglitazone use and bladder cancer risk with long-term use of pioglitazone [22, 23]. As a followup on the former, a safety review of rosiglitazone by a panel of international experts deemed the available data inconclusive and requiring further study. In the latter case, direct clinical evidence of this possible association is also required. Despite the need for more evidence, these drugs remain FDA approved, albeit with warning updates to package inserts clarifying the potential for risk [24, 25], and a Risk Evaluation and Mitigation Strategy (REMS) is in place to restrict access and distribution of rosiglitazone-containing medicines to those healthcare providers and their patients who confirm their awareness of the new warnings [26]. Nevertheless, the utility of these drugs remains valuable not only for their ability to provide mechanistic insight into the role of PPAR*γ*-mediated target regulation, but also for their potential benefit in certain off-label uses.

Dual and pan PPAR ligands were also developed to enhance therapeutic potential via simultaneously activating two or more PPAR isoforms. Examples include PPAR*α*/*γ* modulators like tesaglitazar, muraglitazar, and aleglitazar, and the pan PPAR*α*/(*β*/*δ*)/*γ* agonist chiglitazar [27].

The reported links between the above listed PPAR medicines and their *in vitro* and *in vivo* effects on ABC transporters are summarized in Tables 1 and 2, respectively, and described in detail below in the context of several human diseases.

## 3. Cancer

The goal of chemotherapy is to target rapidly dividing cells or deregulated signaling pathways to suppress tumour growth, and ultimately, cure cancer patients; however, one primary roadblock to the success of chemotherapy is acquisition of multidrug resistance (MDR). A well-known cause of MDR is ABC transporter-driven drug efflux from cancer cells instilling resistance to multiple agents [28]. The well-known ABC transporters, P-glycoprotein (Pgp)/MDR1/ABCB1, multidrug resistance protein (MRP)1/ABCC1, and breast cancer-resistance protein (BCRP)/MXR/ABCG2, are overexpressed in a variety of different human cancers and transport a range of chemotherapeutic drugs [4]. Pgp, an important blood brain barrier component and regulator of intestinal drug absorption, was the first ABC transporter to be characterized in 1976 [29]. Its overexpression in tumours of the kidney, liver, colon, and breast correlates with chemoresistance [30–32]. Substrates of Pgp include anthracyclines, vinca alkaloids, taxanes, camptothecins, mitoxantrone, and methotrexate [33]. The second ABC gene discovered was the more ubiquitously expressed MRP1 [34], which transports anthracyclines, vinca alkaloids, and etoposide, in addition to organic anions and glutathione conjugates [28]. Its overexpression confers chemotherapy resistance in prostate, lung, breast, and neuroblastoma cancer [35, 36]. Finally, BCRP is normally expressed in placenta and small intestine, as well as various stem cell populations [37, 38]. Several drug-resistant cell lines also contain elevated levels of this ABC transporter, which contributes to the efflux of several antitumour agents such as doxorubicin, daunorubicin, mitoxantrone, and topotecan [39–41].

In addition to MDR, other functions of ABC transporters in cancer are beginning to emerge, further implicating these genes as important targets of chemotherapy. For example, Pgp expression, devoid of ATP-dependent drug transport, suppresses cell death in the presence of apoptotic signals in normal and cancer cells [42–44]. Furthermore, Pgp knockdown reduced the migration and invasion potential of MCF7 human breast cancer cells [45]. As a result of these studies, direct inhibition of ABC transporter activity has become an appealing undertaking for researchers in the development of improved cancer chemotherapeutics; however, several clinical trials using ABC inhibitors have proven unsuccessful [46].

Research has shown that PPAR activation induces expression of both mouse (Mdr1/Mdr1b/Abcb1b, Mdr2/Abcb4, and Mdr3/Mdr1a/Abcb1a) and human (MDR2/MDR3/ABCB4) homologs of Pgp, which efflux similar chemotherapy substrates as MDR1 [33]. Fasting-induced fatty acid release increased hepatic expression of Mdr2 mRNA and protein, as well as activity, in wild-type but not PPAR*α*-knockout mice [77]. Similar results were observed in ciprofibrate-treated mice [73]. Interestingly, the latter trial demonstrated that elevated Mdr1 and Mdr3 mRNA expression accompanied Mdr2 induction in liver; however, in cultured mouse hepatocytes, only Mdr2 levels were elevated by PPAR*α* agonists suggesting that *in vivo* induction of Mdr1 and Mdr3 may be influenced by PPAR*α* activation in surrounding tissue. Furthermore, both ciprofibrate and clofibrate increased hepatic expression of Mdr2 mRNA in CF1 mice. This was associated with increased Mdr2 redistribution into bile canaliculi and enhanced biliary phospholipid secretion [78]. Similarly, in a chimeric mouse model with humanized liver, bezafibrate increased hepatic MDR2/MDR3 mRNA and protein, and promoted canalicular localization of the transporter [71]. Bezafibrate-treated HepG2 human hepatocellular liver carcinoma cells also showed elevated expression of MDR2/MDR3 mRNA. Although there was no subsequent change in protein levels, there was a redistribution of the transporter into pseudocanaliculi between cells, accompanied by enhanced apical localization of phospholipids, which could be attenuated by PPAR*α*-specific knockdown [72].

Several MRP1 homologs may also be upregulated by PPARs, including MRP2/ABCC2, MRP3/ABCC3, and MRP4/ABCC4, which are known to transport substrates belonging to a variety of chemotherapy drug classes [33]. Although their normal physiological function remains elusive, it has been suggested that these transporters may play a role in MDR [79, 80]. Additionally, MRP4 expression may play a role in migration, as knockdown or pharmacological inhibition of this transporter appears to prevent human dendritic cell motility [81]. Moffit et al. examined the effect of clofibrate on hepatic transporters in mice. Following 10 days of dosing, clofibrate upregulated hepatic expression of Bcrp, Mrp3, and Mrp4 mRNA and protein in CD1 mice. Similar findings for Mrp3 and Mrp4 were detected in liver tissue isolated from clofibrate-treated wild-type SV129 mice, while no changes were seen in liver from similarly treated PPAR*α*-knockout mice [82]. Liver expression of Mrp3 was also induced in C57BL mice treated with clofibrate, ciprofibrate, and diethylhexyl phthalate (DEHP) [83]. Maher et al. also reported the hepatic induction of Mrp3 and Mrp4 transcription in perfluorodecanoic-acid- (PFDA-) treated mice [84]. This was associated with elevated serum levels of serum-conjugated bilirubin and bile acids indicative of Mrp3- and Mrp4-specific hepatic efflux activity. These effects were attenuated in PPAR*α*-knockout mice treated with PFDA. Several putative PPRE sequences were identified upstream of the Mrp3 and Mrp4 promoters, providing further evidence that PPAR*α* may directly regulate transcription of these transporters in the liver.

Activation of PPARs may also induce expression of BCRP. PPAR*α* agonists upregulate Bcrp transcription in mouse intestine [85]. Furthermore, PPAR*α*-dependent activation induces BCRP expression and efflux activity in human cerebral endothelial cells [75]. Here, transporter induction is accompanied by binding of PPAR*α* to a PPRE within the BCRP promoter. In human monocyte-derived dendritic cells, BCRP was directly induced by ligand-activated PPAR*γ* through three functional PPRE sequences located within the gene's promoter [76]. This enhancement of BCRP activity elevated drug efflux and maintained intracellular low levels of mitoxantrone, which could be reversed by addition of a BCRP inhibitor. In doxorubicin-resistant MCF7 breast cancer and K562 human leukemia cell lines, troglitazone downregulated expression of BCRP, and restored sensitivity to doxorubicin treatment [69]. Although troglitazone may elicit effects that are PPAR*γ*-dependent, it is also known to operate via pathways that are independent of this nuclear receptor [86]. Inhibition of PPAR*γ* in untreated MCF7 cells reduced BCRP expression indicating that the observed effects of troglitazone were PPAR*γ*-independent, and providing evidence that this TZD may suppress BCRP transcription in these cells by indirectly antagonizing PPAR*γ* itself.

In contrast to the studies previously outlined, a number of reports indicate that PPAR activation may inhibit ABC transporter expression and activity. Chen et al. observed that troglitazone increased PPAR*γ* activity and reversed Pgp-mediated chemoresistance in vincristine-resistant SGC7901 human gastric cancer cells [70]. Furthermore, Rajkumar and Yamuna performed genetic expression analysis on a doxorubicin-resistant 143B human osteosarcoma cell line and found increased expression of Pgp and Kruppel-like factor 2 [91]. Given that the latter is a known suppressor of PPAR*γ* expression [92], these findings may implicate the PPAR*γ* pathway as a negative regulator of Pgp transcription. Wang et al. also demonstrated that tumour necrosis factor (TNF)*α* could partially reverse MDR by inducing PPAR*α* and suppressing Pgp in an adriamycin-resistant cell line derived from HepG2 cells [93]. In another study, PPAR*α* agonists downregulated Mrp1 expression in mouse intestine [85]. Hepatic expression of Mrp2 protein was reduced in male Sprague-Dawley rats treated with the PPAR*α* agonists, clofibrate, DEHP, and PFDA [89]. Furthermore, efflux of bile acids by Mrp2 may be suppressed by troglitazone in cultured rat hepatocytes [74]. Both rosiglitazone and troglitazone inhibited BCRP function in BCRP-overexpressing MDCKII canine kidney epithelial cells, but induced its transcription in the HuH7 human hepatoma cell line [68]. These PPAR*γ* activators also decreased Pgp-mediated drug efflux in doxorubicin-resistant P388 mouse leukemia cells. Moreover, fenofibrate suppressed Mdr1 transport activity in L-MDR1 porcine kidney epithelial cells [67]. Finally, in doxorubicin-resistant MCF7 and K562 cells, troglitazone downregulated expression of Pgp and reversed chemoresistance to doxorubicin [69]. However, among these studies it was not clarified if these activities were dependent on PPAR activation and signaling.

From the laboratory perspective, the involvement of ABC transporters in MDR and other cancer hallmarks necessitate these genes as vital targets of chemotherapy, whereas their precise role in the clinical manifestation of cancer remains elusive. This is likely why clinical trials with Pgp inhibitors failed to reduce drug efflux and subsequent chemoresistance [94]. Regulation of ABC gene transcription by PPARs may be another option, but primarily, a detailed understanding of the functional and clinical relevance of the entire ABC transporter family in tumour samples and cell lines is obligatory. Future studies may identify new roles for ABC transporters in cancer, which could be targeted by either pharmacological inhibition or regulation of PPARs. Most of the evidence implies that PPARs are positive regulators of cancer-related ABC genes, indicating that transporter expression can be suppressed by antagonizing PPARs. On the other hand, controversial findings have also been reported; therefore, improved understanding of the mechanism by which PPARs regulate ABC genes is required. In particular, delineating the effects of PPAR-dependent and -independent signaling on ABC gene transcription will determine the precise link between PPARs and ABC transporters in cancer and may predict the success of PPAR ligand therapy in reversing MDR. Additional studies exploring the effect of PPAR activation as an adjuvant to chemotherapy in a wide range of drug-resistant cancer cell lines may also prove insightful.

## 4. Atherosclerosis

The atherosclerotic condition is characterized by the thickening of arterial vessels as a result of an accumulation of oxidized low-density lipoproteins (LDL), and subsequently, cholesterol-laden macrophages as a consequence of a maladaptive immune response. The associated chronic inflammation and necrosis drives plaque formation and vessel hardening, which can invariably lead to coronary artery disease (CAD)—the leading cause of death worldwide [95]. Interestingly, recent evidence suggests that PPAR induction of ABC transporter expression may improve lipid profiles through enhanced cholesterol cycling and excretion, and thus represents a promising avenue to prevent cardiovascular disease progression.

As noted above, PPAR*α* and PPAR*γ* isoforms are also expressed in immune cells, such as mature macrophages, where they regulate genes involved in inflammation, differentiation, and TNF-*α*/IFN-*γ*-mediated apoptosis [96–98]. Expression of these two PPAR isoforms is also observed in macrophage foam cells that constitute atherosclerotic lesions [20, 99–101]. Recent studies suggest activating PPARs exerts antiatherosclerotic properties via improved cholesterol homeostasis through the regulation of specific ABC transporters. ABCA1 is one such transporter that controls apolipoprotein-A1- (apoA1-) mediated cholesterol efflux in macrophages [102]. Another, ABCG1, also promotes the transport of cholesterol from macrophages to HDL, although the underlying mechanism remains unclear [103]. This efflux is a critical step in reverse cholesterol transport, a process that allows for cholesterol displacement and excretion by the liver, and represents a protective modality against atherosclerotic risk.

Activation of PPAR*γ* stimulates apoA1-mediated cholesterol efflux from human and mouse macrophages and foam cells through a signaling cascade that culminates in ABCA1 induction [52, 57, 62]. This activity is mediated via PPAR*γ*-dependent induction of liver X receptor (LXR*α*), an oxysterol-activated nuclear receptor, that triggers ABCA1 transcription via interaction with specific response elements in the ABCA1 promoter [104]. Although several putative PPRE sequences were initially identified in the LXR*α* promoter [105], only one was confirmed as a preferential PPAR*γ* binding site in macrophages [57]. In addition, specific ligands for PPAR*α*, PPAR*β*/*δ*, and PPAR*γ* all increase LXR*α* and ABCA1 mRNA and protein and enhance apoA1-mediated lipid efflux and HDL synthesis in THP1 macrophages, suggesting that non-PPRE-dependent regulatory mechanisms may be responsible for some of these activities [47, 51]. In a similar study, THP1 macrophages treated with various PPAR ligands revealed that PPAR*β*/*δ* activation induced greater ABCA1 mRNA expression and apoA1-mediated cholesterol efflux compared to PPAR*α* and PPAR*γ* agonists [61]. Both rosiglitazone and pioglitazone treatment of THP1 macrophages also stimulated cholesterol efflux and induced ABCA1 mRNA and protein expression, implicating a regulatory role for PPAR*γ* [56, 58, 59]. Correspondingly, treatment of mouse RAW264.7 macrophage-derived foam cells with conjugated linoleic acid (CLA) isomers (*c*9*t*11-CLA and *t*10*c*12-CLA) or the hydroxylated derivative of linoleic acid (13-HODE), known ligands of both PPAR*α* and PPAR*γ*, decreased cholesterol accumulation, enhanced cholesterol clearance, and induced expression of Abca1, and other genes involved in cholesterol homeostasis [54, 55]. Similarly, in other tissues, such as canine gallbladder epithelial cells, and human mesangial and skeletal muscle cells, PPAR activators upregulate LXR*α*-mediated ABCA1 transcription and prevent cholesterol accumulation [48, 53, 64].

Another PPAR*γ* activator, telmisartan, induced Abca1 and Abcg1 expression in murine macrophages, and in the aorta of ApoE-deficient mice, where it suppressed macrophage proliferation and atherosclerotic progression [63]. It was also reported that the conditional deletion of PPAR*γ* in macrophages led to decreased expression of LXR*α*, Abcg1, and ApoE in mice [106]. This was accompanied by a significant reduction in cholesterol efflux from macrophages to HDL. Furthermore, granulocyte macrophage colony-stimulating factor (GM-CSF) knockout mice showed reduced expression of PPAR*γ* and Abcg1 in alveolar macrophages of the lung. Given that GM-CSF is a known positive regulator of PPAR*γ*, reintroduction of PPAR*γ* in alveolar macrophages increased Abcg1 expression and cholesterol efflux activity and decreased intracellular lipid content [107]. Consequently, PPAR*γ* activation by pioglitazone induced cholesterol efflux activity and increased ABCG1 mRNA and protein in THP1 and RAW264.7 macrophages [56]. Fenofibrate also stimulated Abcg1 transcription, which was associated with increased HDL particle size, in Zucker diabetic fatty rats [90].

In the liver, ABCA1 is implicated in control of HDL synthesis, which represents another means of protecting against atherosclerosis. HDLs are specialized carrier molecules in the blood that transport cholesterol from peripheral tissues and cholesterol-laden macrophages to the liver for excretion [108]. This process is thought to be the main mechanism underlying HDL's antiatherosclerotic properties [109]. Indeed, plasma HDL levels correspond inversely with cardiovascular risk [110]. Consequently, impaired ABCA1 activity is associated with low plasma HDL, which is linked to Tangier disease, familial HDL deficiency, and accelerated atherosclerosis [111]. Furthermore, Abcg1-overexpressing transgenic mice have greater plasma HDL levels, improved cholesterol efflux from macrophages, and reduced atherosclerotic burden [112].

Several studies have demonstrated the ability of PPARs to regulate ABCA1 expression in the liver. In one study, PPAR activation with a variety of fibrates upregulated LXR*α* expression coupled with enhanced ABCA1 transcription and HDL biosynthesis in HepG2 cells [49]. Of the fibrates used, fenofibrate and LY518674 acted exclusively through PPAR*α*, while bezafibrate and gemfibrozil preferred PPAR*γ* and PPAR*β*/*δ*, respectively, in addition to PPAR*α* activity. Accordingly, antagonism of PPAR*γ* in HepG2 cells blocked upregulation of ABCA1 mRNA and protein; however, PPAR*γ* activation also reduced ABCA1 protein levels in this cell line despite increased ABCA1 transcription [60]. In this model, activation of PPAR*γ* caused the dissociation of LXR*β* from ABCA1 at the cell membrane leading to increased ABCA1 protein degradation. Subsequently, translocation of LXR*β* to the nucleus increased ABCA1 transcription via binding of this nuclear receptor to the promoter region of the ABCA1 gene. Whether this affected HDL biosynthesis or cholesterol efflux from HepG2 cells remains to be seen.

Fasting-associated fatty acid release induces hepatic expression of Abca1, Abcg5, and Abcg8 in wild-type but not PPAR*α*-null mice [77]. Although these ABC transporters are involved in hepatobiliary cholesterol transport, maximal cholesterol excretion from the liver was decreased by ~50% after fasting. This raises the possibility of other PPARs and PPAR agonists playing a role in ABC transporter-mediated liver cholesterol efflux under normal conditions. More recently, a clinical trial examined the effect of fenofibrate treatment on HDL subclass particle concentrations on patients with triglycerides ≥150 mg/dL [87]. Following 3 weeks of therapy, stratification of participants by ABCA1 polymorphism genotypes revealed two variants (R1587K and R219K) that were associated with significant increases in small HDL particles. This suggests a synergism between ABCA1 polymorphism and PPAR*α* agonists.

One of the most intuitive ways to reduce the burden of atherosclerosis is to regulate the uptake of dietary cholesterol at the intestine. In mice, intestinal expression of Abca1 and Abcg8 is induced upon fasting [113]. Furthermore, normal mice maintained on a diet supplemented with a PPAR*α* activator showed an increase in intestinal Abca1 gene transcription and protein compared to PPAR*α*-deficient mice, which showed no effect to treatment [88]. This increased expression was associated with a reduction in cholesterol absorption, as well as decreased plasma and liver cholesterol concentrations.

Atherosclerotic heart disease is undoubtedly one of the most devastating diseases worldwide. While pharmacological and dietary interventions that lower LDL levels remain the current treatment paradigm for atherosclerosis, they may only decrease the incidence of cardiovascular events by ~30% [109]. The literature indicates that induction of ABCA1 and ABCG1 expression by PPAR activation may play a role in preventing atherosclerosis by improving cholesterol homeostasis and HDL synthesis. Moving forward, additional studies are required to address the clinical significance of these activities and to determine whether or not they are PPAR dependent. Clinical trials have begun to examine the effect of some PPAR activators in atherosclerosis, yielding a mixture of results. For example, fenofibrate treatment barely increased HDL levels and marginally lowered the incidence of CAD in high-risk patients with type 2 diabetes [114, 115]. In a similar study, gemfibrozil significantly reduced CAD, in part, by elevating HDL [116]. Studies have also demonstrated that TZDs promote the destabilization of atherosclerotic plaques in nondiabetic patients [117], while still others report that these PPAR activators may actually increase the risk of heart failure in type 2 diabetics [118]. Despite these findings, a better understanding of the pleiotropic effects of PPARs and their role in atherosclerosis is required in order to design and develop appropriate PPAR-based therapies devoid of detrimental effects.

## 5. Ichthyosis

Derived from the Greek *ichthys* for “fish,” ichthyosis refers to a group of dermatological disorders generally described by severely dry, cracked, and flaky skin that is thought to bear resemblance to fish scales [119]. The main pathophysiological feature of this disease is a failure of skin barrier permeability, leading to a spectrum of conditions ranging from the most mild, such as the common ichthyosis vulgaris, to the most severe, such as Harlequin type ichthyosis, which is rare but fatal in newborns. Recently, mutations in ABCA12, a keratinocyte lipid transporter, were shown to underlie the latter phenotype [120, 121]. Under normal conditions, ABCA12 facilitates the uptake of lipids into specialized secretory granules, called lamellar bodies, within keratinocytes. These lipid-filled granules are then liberated from the cell where they release their cargo to the outermost layer of the epidermis, a requirement for normal formation of skin barrier permeability. On the other hand, ABCA12 deficiency prevents lipid loading into lamellar bodies, which leads to abnormal development of the skin and strikingly elevated rates of prenatal mortality [122].

While studies in this area are limited, they have demonstrated that ABCA12 may be regulated by PPARs, which may have important implications in Harlequin ichthyosis. Activation of PPARs promotes lamellar body secretion and improved epidermal barrier permeability in mice [123]. More recently, Jiang et al. demonstrated that ciglitazone, troglitazone, and the PPAR*β*/*δ* agonist, GW610742, induced expression of ABCA12 mRNA and protein in human keratinocytes [65]. Similarly, ceramide-induced transcription of ABCA12 was attenuated by siRNA knockdown of PPAR*β*/*δ*, indicating that this activity was dependent on PPAR*β*/*δ* [66]. In a separate experiment, Jiang et al. also demonstrated that clofibrate and the PPAR*β*/*δ* ligand, GW501516, increased expression of the ABCA1 cholesterol efflux pump in human keratinocytes [50]. Given that these cells require cholesterol for adequate formation of permeability barrier function [124], ABCA1 regulation by PPARs may also play an important role in understanding the pathophysiology of Harlequin ichthyosis. These findings implicate the potential utility of PPAR ligands for the treatment of this disease, which should be further validated *in vivo*.

## 6. Conclusion

These studies describe compelling evidence for PPAR medicines in the regulation of ABC transporter expression and function. Beyond their respective individual roles in various human diseases, the overlap in tissue distribution and regulatory potential between PPARs and certain ABC transporters make this emerging story an attractive field for further research. They also provide an alternative approach when the targeting of ABC transporter genes in human cancer, atherosclerosis, or ichthyosis may suggest therapeutic advantages for patients. In addition, targeting ABC transporters at the transcriptional level may circumvent issues previously identified during focused inhibition of transporter activity. Furthermore, given the complex and multistage etiology of cancer and atherosclerosis, dual/pan PPAR modulators may prove especially useful in simultaneously regulating multiple PPAR isoforms and ABC transporters. For example, examining PPAR*α*/*γ* agonists like aleglitazar, currently being assessed for cardiovascular safety in Phase 3 clinical trials, for synergistic effects on multiple ABC transporters may prove a fruitful area for future studies. Improving our understanding of the interactions between PPARs, their ligands, and ABC transporters will further aid in developing more targeted therapeutic strategies to mitigate the burden of human disease on patients and the healthcare system.

## Figures and Tables

**Table 1:** *In vitro* effects of PPAR ligands on ABC transporters.

ABC transporter	PPAR	PPAR Ligand	Cell line	Transporter effect	Reference
*ABCA1*	PPAR*α*	Bezafibrate	Primary mouse fibroblasts	↑ ABCA1 and LXR*α* mRNA	[47]
			THP1 human macrophages	↑ apoA1-mediated cholesterol efflux	
			WI38 human fibroblasts		
					
			Immortalized human mesangial cells	↑ ABCA1 and LXR*α* mRNA	[48]
				↑ apoA1-mediated cholesterol efflux	
					
			Primary mouse hepatocytes	↑ ABCA1 mRNA and protein	[49]
			HepG2 human hepatoma cells	↑ HDL synthesis	
					
		Clofibrate	Primary human foreskin keratinocytes	↑ ABCA1 mRNA	[50]
					
		Fenofibrate	Primary mouse fibroblasts	↑ ABCA1 and LXR*α* mRNA	[47]
			THP1 human macrophages	↑ apoA1-mediated cholesterol efflux	
			WI38 human fibroblasts		
					
			BALB/3T3 mouse fibroblasts	↑ ABCA1 mRNA and protein	[51]
			RAW264.7 mouse leukemic macrophages	↑ apoA1-mediated cholesterol efflux	
			THP1 human macrophages		
					
			Primary mouse hepatocytes	↑ ABCA1 mRNA and protein	[49]
			HepG2 human hepatoma cells	↑ HDL synthesis	
					
		Gemfibrozil	Primary mouse fibroblasts	↑ ABCA1 and LXR*α* mRNA	[47]
			THP1 human macrophages	↑ apoA1-mediated cholesterol efflux	
			WI38 human fibroblasts		
					
			Primary mouse hepatocytes	↑ ABCA1 mRNA and protein	[49]
			HepG2 human hepatoma cells	↑ HDL synthesis	
					
		LY518674	Primary mouse fibroblasts	↑ ABCA1 and LXR*α* mRNA	[47]
			THP1 human macrophages	↑ apoA1-mediated cholesterol efflux	
			WI38 human fibroblasts		
					
			Primary mouse hepatocytes	↑ ABCA1 mRNA and protein	[49]
			HepG2 human hepatoma cells	↑ HDL synthesis	
					
		RPR-5	Primary human macrophages	↑ ABCA1 and LXR*α* mRNA	[52]
					
		WY14643	Immortalized human mesangial cells	↑ ABCA1 and LXR*α* mRNA	[48]
				↑ apoA1-mediated cholesterol efflux	
					
			Primary human macrophages	↑ ABCA1 and LXR*α* mRNA	[52]
				↑ apoA1-mediated cholesterol efflux	
					
			THP1 human macrophages	↑ ABCA1 mRNA	[52]
					
			BALB/3T3 mouse fibroblasts	↑ ABCA1 mRNA and protein	[51]
			RAW264.7 mouse leukemic macrophages	↑ apoA1-mediated cholesterol efflux	
			THP1 human macrophages		
					
			Primary canine gallbladder epithelial cells	↑ ABCA1 mRNA and protein	[53]
					
	PPAR*α*/*γ*	13-HODE	RAW264.7 mouse leukemic macrophages	↑ Abca1 and LXR*α* protein	[54]
				↑ cholesterol efflux	
					
		*c*9*t*11-CLA	RAW264.7 mouse leukemic macrophages	↑ Abca1 mRNA and protein	[55]
				↑ LXR*α* mRNA	
				↑ HDL-mediated cholesterol efflux	
					
		t10c12-CLA	RAW264.7 mouse leukemic macrophages	↑ Abca1 mRNA and protein	[55]
				↑ LXR*α* mRNA	
				↑ HDL-mediated cholesterol efflux	
					
		NO-pravastatin	Primary canine gallbladder epithelial cells	↑ ABCA1 mRNA and protein	[53]
				↑ LXR*α* mRNA	
					
		Pravastatin	Primary canine gallbladder epithelial cells	↑ ABCA1 mRNA and protein	[53]
				↑ LXR*α* mRNA	
					
		Simvastatin	Primary canine gallbladder epithelial cells	↑ ABCA1 mRNA and protein	[53]
				↑ LXR*α* mRNA	
					
	PPAR*γ*	Pioglitazone	Primary mouse fibroblasts	↑ ABCA1 and LXR*α* mRNA	[47]
			THP1 human macrophages	↑ apoA1-mediated cholesterol efflux	
			WI38 human fibroblasts		
					
			RAW264.7 mouse leukemic macrophages	↑ Abca1 mRNA and protein	[56]
			THP1 human macrophages	↑ cholesterol efflux	
					
		Rosiglitazone	Primary human macrophages	↑ ABCA1 and LXR*α* mRNA	[52]
				↑ apoA1-mediated cholesterol efflux	
					
			THP1 human macrophages	↑ ABCA1 mRNA	[52]
					
				↑ ABCA1 and LXR*α* mRNA	[57]
				↑ cholesterol efflux	
					
				↑ ABCA1 mRNA and protein	[58]
					
				↑ ABCA1 mRNA and protein	[59]
				↓ intracellular cholesterol	
					
		Troglitazone	Primary human macrophages	↑ ABCA1 and LXR*α* mRNA	[52]
					
			THP1 human macrophages	↑ ABCA1 mRNA	[52]
					
			Primary canine gallbladder epithelial cells	↑ ABCA1 mRNA and protein	[53]
					
		GW1929	HepG2 human hepatoma cells	↑ ABCA1, LXR*α*, and LXR*β* mRNA	[60]
				↓ ABCA1 and LXR*β* protein	
					
		GW7845	THP1 human macrophages	↑ ABCA1 mRNA	[61]
					
		Mycophenolic acid	HepG2 human hepatoma cells	↑ ABCA1 mRNA and protein	[62]
				↑ LXR*α* protein	
					
		Prostaglandin J2	Immortalized human mesangial cells	↑ ABCA1 and LXR*α* mRNA	[48]
				↑ apoA1-mediated cholesterol efflux	
					
			Primary human macrophages	↑ ABCA1 and LXR*α* mRNA	[52]
					
		Telmisartan	RAW264.7 mouse leukemic macrophages	↑ Abca1 mRNA	[63]
				↓ macrophage proliferation	
					
	PPAR*β*/*δ*	GW501516	Primary mouse fibroblasts	↑ ABCA1 and LXR*α* mRNA	[47]
			THP1 human macrophages	↑ apoA1-mediated cholesterol efflux	
			WI38 human fibroblasts		
					
			THP1 human macrophages	↑ ABCA1 mRNA	[61]
			1BR3N human fibroblasts	↑ apoA1-mediated cholesterol efflux	
					
			FHS74 human intestinal cells	↑ ABCA1 mRNA	[61]
					
			Primary human skeletal muscle cells	↑ ABCA1 mRNA	[64]
					
			Primary human foreskin keratinocytes	↑ ABCA1 mRNA	[50]

*ABCA12*	PPAR*γ*	Ciglitazone	Primary human foreskin keratinocytes	↑ ABCA12 mRNA and protein	[65]
					
		Troglitazone	Primary human foreskin keratinocytes	↑ ABCA12 mRNA	[65]
					
		GI251929X	Primary human foreskin keratinocytes	↑ ABCA12 mRNA	[65]
					
	PPAR*β*/*δ*	Ceramide	Primary human foreskin keratinocytes	↑ ABCA12 mRNA and protein	[66]
					
		GW610742	Primary human foreskin keratinocytes	↑ ABCA12 mRNA and protein	[65]

*Pgp/MDR1/ABCB1*	PPAR*α*	Fenofibrate	Pgp-overexpressing L-MDR1 porcine kidney epithelial cells	↓ calcein efflux	[67]
					
	PPAR*α*/*γ*	Simvastatin	Pgp-overexpressing L-MDR1 porcine kidney epithelial cells	↓ calcein efflux	[67]
					
	PPAR*γ*	Rosiglitazone	Doxorubicin-resistant P388 mouse leukemia cells	↓ calcein efflux	[68]
					
		Troglitazone	Doxorubicin-resistant P388 mouse leukemia cells	↓ calcein efflux	[68]
					
			Doxorubicin-resistant K562 human leukemia cells	↓ Pgp protein	[69]
			Doxorubicin-resistant MCF7 human breast cancer cells	↑ sensitivity to doxorubicin	
					
			Vincristine-resistant SGC7901 human gastric cancer cells	↓ Pgp mRNA and protein	[70]
				↓ Rh123 efflux	
				↑ sensitivity to vincristine	

*MDR2/MDR3/ABCB4*	PPAR*α*	Bezafibrate	HepG2 human hepatoma cells	↑ MDR2/MDR3 mRNA	[71]
				↑ MDR2/MDR3 redistribution	
					
				↑ MDR2/MDR3 mRNA	[72]
				↑ MDR2/MDR3 redistribution	
				↑ phospholipid efflux	
					
		Ciprofibrate	Primary mouse hepatocytes	↑ Mdr2 mRNA	[73]
					
		WY14643	Primary mouse hepatocytes	↑ Mdr2 mRNA	[73]

*MRP2/ABCC2*	PPAR*γ*	Troglitazone	Primary rat hepatocytes	↓ Mrp2-associated bile efflux	[74]

*ABCG1*	PPAR*α*/*γ*	13-HODE	RAW264.7 mouse leukemic macrophages	↑ Abcg1 and LXR*α* protein	[54]
				↑ cholesterol efflux	
					
	PPAR*γ*	Pioglitazone	RAW264.7 mouse leukemic macrophages	↑ ABCG1 mRNA and protein	[56]
			THP1 human macrophages	↑ cholesterol efflux	
					
		Rosiglitazone	THP1 human macrophages	↑ ABCG1 and LXR*α* mRNA	[57]
				↑ cholesterol efflux	
					
		Telmisartan	RAW264.7 mouse leukemic macrophages	↑ Abcg1 mRNA	[63]
				↓ macrophage proliferation	

*BCRP/ABCG2*	PPAR*α*	Clofibrate	HCMEC/D3 human cerebral microvascular endothelial cells	↑ BCRP mRNA and protein	[75]
				↑ mitoxantrone efflux	
					
		GW7647	HCMEC/D3 human cerebral microvascular endothelial cells	↑ BCRP mRNA and protein	[75]
					
	PPAR*γ*	Rosiglitazone	Primary human dendritic cells	↑ BCRP mRNA and protein	[76]
				↑ Hoescht efflux	
				↑ mitoxantrone efflux	
				↑ sensitivity to mitoxantrone	
					
			BCRP-overexpressing MDCKII canine kidney epithelial cells	↓ PhA efflux	[68]
					
			HuH7 human hepatoma cells	↑ BCRP mRNA	[68]
					
		Troglitazone	Primary human dendritic cells	↑ BCRP mRNA	[76]
					
			HuH7 human hepatoma cells	↑ BCRP mRNA	[68]
					
			Doxorubicin-resistant K562 human leukemia cells	↓ BCRP protein	[69]
			Doxorubicin-resistant MCF7 human breast cancer cells	↑ sensitivity to doxorubicin	
					
	GW7845		Primary human dendritic cells	↑ BCRP mRNA	[76]
					
	GW9662		Doxorubicin-resistant MCF7 human breast cancer cells	↓ BCRP protein	[69]

**Table 2:** *In vivo* effects of PPAR ligands on ABC transporters.

ABC transporter	Ligand	Receptor	Model	Transporter effect	Reference
*ABCA1*	PPAR*α*	Fenofibrate	Hypertriglyceridemic patients	Differential HDL synthesis due to ABCA1 variants	[87]
					
		WY14643	SV129 mice	↑ Abca1 mRNA and protein in intestine	[88]
				↓ intestinal absorption of cholesterol	
					
	PPAR*γ*	Telmisartan	ApoE−/− C57BL mice	↑ Abca1 mRNA in aorta	[63]
				↓ atherosclerotic lesion size and number	

*Pgp/MDR1/ABCB1*	PPAR*α*	Ciprofibrate	SV129 mice	↑ hepatic Mdr1 & Mdr3 mRNA	[73]

*MDR2/MDR3/ABCB4*	PPAR*α*	Bezafibrate	CF1 mice	↑ hepatic Mdr2 mRNA	[78]
				↑ bile secretion of phospholipid	
					
			Humanized liver-uPA/SCID chimeric mice	↑ hepatic MDR2/MDR3 mRNA and protein	[71]
				↑ hepatic MDR2/MDR3 redistribution into bile canaliculi	
					
		Ciprofibrate	SV129 mice	↑ hepatic Mdr2 mRNA and protein	[73]
				↑ bile secretion of cholesterol and phospholipids	
					
			CF1 mice	↑ hepatic Mdr2 mRNA	[78]
				↑ Mdr2 redistribution into bile canaliculi	
				↑ bile secretion of phospholipid	
					
		Clofibrate	CF1 mice	↑ hepatic Mdr2 mRNA	[78]
				↑ Mdr2 redistribution into bile canaliculi	
				↑ bile secretion of phospholipid	
					
		Fenofibrate	CF1 mice	↑ hepatic Mdr2 mRNA	[78]
					
		Gemfibrozil	CF1 mice	↑ hepatic Mdr2 mRNA	[78]

*MRP1/ABCC1*	PPAR*α*	Ciprofibrate	C57BL mice	↓ hepatic Mrp1 mRNA	[83]
					
		Clofibrate	C57BL mice	↓ hepatic Mrp1 mRNA	[83]
					
		GW7647	C57BL mice	↓ Mrp1 mRNA in small intestine	[85]
					
		WY14643	C57BL mice	↓ Mrp1 mRNA in small intestine	[85]

*MRP2/ABCC2*	PPAR*α*	Clofibrate	Sprague-Dawley rats	↓ hepatic Mrp2 protein	[89]
					
		DEHP	Sprague-Dawley rats	↓ hepatic Mrp2 protein	[89]
					
		PFDA	Sprague-Dawley rats	↓ hepatic Mrp2 protein	[89]

*MRP3/ABCC3*	PPAR*α*	Ciprofibrate	C57BL mice	↑ hepatic Mrp3 mRNA	[83]
					
		Clofibrate	C57BL mice	↑ hepatic Mrp3 mRNA	[83]
					
			CD1 mice	↑ hepatic Mrp3 mRNA and protein	[82]
			SV129 mice		
					
		DEHP	C57BL mice	↑ hepatic Mrp3 mRNA	[83]
					
		PFDA	C57BL mice	↑ hepatic Mrp3 mRNA	[84]
				↑ serum levels of bilirubin and bile acids	

*MRP4/ABCC4*	PPAR*α*	Clofibrate	CD1 mice	↑ hepatic Mrp4 mRNA and protein	[82]
			SV129 mice		
					
		PFDA	C57BL mice	↑ hepatic Mrp3 mRNA	[84]
				↑ serum levels of bilirubin and bile acids	

*ABCG1*	PPAR*α*	Fenofibrate	Zucker diabetic fatty rats	↑ Abcg1 mRNA	[90]
				↑ HDL particle size	
					
	PPAR*γ*	Telmisartan	ApoE−/− C57BL mice	↑ Abcg1 mRNA in aorta	[63]
				↓ atherosclerotic lesion size and number	

*BCRP/ABCG2*	PPAR*α*	Clofibrate	CD1 mice	↑ hepatic Bcrp mRNA and protein	[82]
					
			SV129 mice	↑ hepatic Bcrp mRNA	[82]
					
		GW7647	C57BL mice	↑ Bcrp mRNA in small intestine	[85]
					
		WY14643	C57BL mice	↑ Bcrp mRNA in small intestine	[85]
